# Dermatomyositis-like Eruptions, Hydroxyurea-Associated Squamous Dysplasia, and Nonmelanoma Skin Cancer: A Case Report and Systematic Review

**DOI:** 10.3390/dermatopathology12020011

**Published:** 2025-03-30

**Authors:** Giorgia Di Marco, Gianmarco Diego Bigotto, Eleonora Cossar, Nathalie Rizzo, Stefania Guida, Franco Rongioletti

**Affiliations:** 1 Dermatology Clinic, IRCCS San Raffaele Hospital, 20132 Milan, Italy; dimarco.giorgia1@hsr.it (G.D.M.);; 2 School of Medicine, Vita-Salute San Raffaele University, 20132 Milan, Italy; e.cossar@studenti.unisr.it; 3 Pathology Unit, IRCCS San Raffaele Hospital, 20132 Milan, Italy

**Keywords:** hydroxyurea-induced dermatitis, dermatomyositis-like eruption, hydroxyurea-associated squamous dysplasia, hydroxyurea-associated squamous cell carcinoma, p53 overexpression

## Abstract

Hydroxyurea (HU), a cornerstone treatment for myeloproliferative disorders, is associated with a wide range of cutaneous side effects, from xerosis and hyperpigmentation to more severe conditions like dermatomyositis-like eruptions (DM-LE) and nonmelanoma skin cancers (NMSC), particularly squamous cell carcinoma (SCC). In this review, we present a unique case of HU-induced DM-LE with histological evidence of keratinocyte dysplasia and p53 overexpression, followed by a systematic analysis of similar cases. Our findings reveal that the clinical presentation of DM-LE, while typically considered benign, shares clinical and histological features with hydroxyurea-associated squamous dysplasia (HUSD), a precancerous condition that may progress to SCC in chronically exposed patients. Key insights include the characteristic histopathological findings of DM-LE, the role of chronic HU therapy and UV-induced damage in promoting p53 overexpression, and the overlap between DM-LE and HUSD. Regular dermatologic monitoring, patient education on photoprotection, and the careful assessment of skin lesions in long-term HU users are essential for the early detection and prevention of malignancies. This review underscores the importance of distinguishing between DM-LE, HUSD, and SCC to optimize management and minimize risks associated with HU therapy.

## 1. Introduction

Hydroxyurea (HU) is a widely used antimetabolite drug for treating myeloproliferative disorders. While it is generally well tolerated, a significant proportion of patients (11–36%) experience cutaneous side effects. These include facial erythema, hyperpigmentation, xerosis, alopecia, skin atrophy, melanonychia, and lower limb ulcers. Less common but clinically significant manifestations, such as dermatomyositis-like eruptions (DM-LE) and nonmelanoma skin cancer (NMSC), have also been reported [1].

A unique presentation associated with chronic HU therapy involves photodistributed erythematous patches and xerosis, resembling photodermatitis or DM-LE. Histologically, these conditions are characterized by nuclear atypia and p53 expression, suggesting a potential premalignant state. This has led to the introduction of the term “hydroxyurea-associated squamous dysplasia” (HUSD) to describe this precancerous condition [2,3,4].

In this paper, we present a patient under long-term therapy with HU who developed a DM-LE with histological features of dermatomyositis and keratinocyte dysplasia, characterized by p53 overexpression. Additionally, we conduct a systematic review of similar cases to further elucidate the clinical and histological characteristics of DM-LE, HUSD, and HU-associated NMSC. This review highlights their potential interrelationships and underscores the importance of vigilant, long-term monitoring for patients undergoing HU therapy.

## 2. Case Report

**Figure 1:**
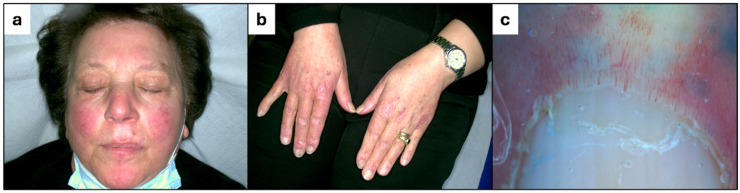
(**a**) Erythema of the face. (**b**) Gottron-like papules on the interphalangeal and metacarpophalangeal joints of the hands. (**c**) Dilated capillaries and architectural disarray of the nailfolds.

A 70-year-old woman with a history of polycythemia vera, treated with hydroxyurea since 2011, presented with a 6-month history of erythematous rash on the face and violaceous papules on the dorsa of her hands. Her medical history also included Raynaud’s phenomenon since 2020, multinodular goiter, and atrial fibrillation. On examination, red-violaceous papules were observed overlying the interphalangeal and metacarpophalangeal joints of the hands (Figure 1b), while the malar region of the face displayed erythema and the eyebrow region exhibited diffuse actinic keratosis (Figure 1a). The dermoscopic evaluation of the nailfolds revealed dilated capillaries and architectural disarray (Figure 1c).

**Figure 2:**
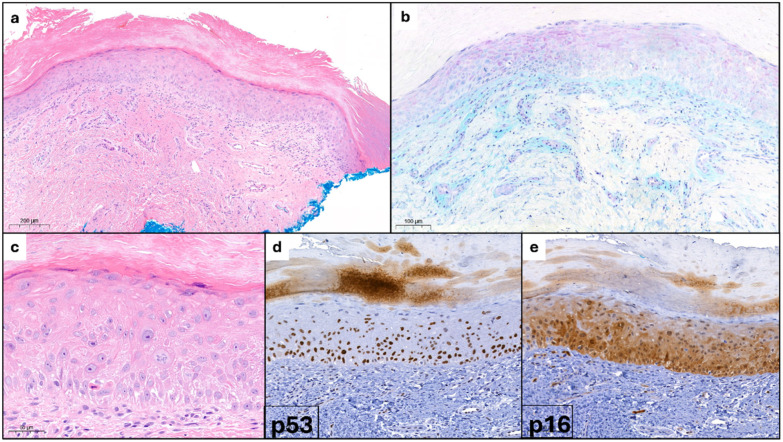
(**a**) Orthokeratotic hyperkeratosis with mild epidermal atrophy; angioplasia and vascular ectasia with mild lymphocytic infiltrate. (**b**) Mucin with Alcian-PAS staining. (**c**) Keratinocyte atypia, dyskeratosis, and nuclear irregularities. (**d**) p53 positivity. (**e**) p16 positivity.

Laboratory exams showed positive antinuclear antibodies (ANAs) at a titer of 1:640 with a homogeneous pattern and elevated erythrocyte sedimentation rate (ESR) at 44 mm/h. A biopsy of the affected skin of the hand was performed and the histologic examination revealed the presence of orthokeratotic hyperkeratosis with slight epidermal atrophy, superficial angioplasia, and a mild perivascular lymphocytic infiltrate (Figure 2a), along with occasional mucin deposits, as detected by Alcian-PAS staining (Figure 2b). At higher magnification keratinocyte atypia, dyskeratosis and nuclear irregularities were also seen (Figure 2c). Immunohistochemical staining showed p53 positivity in the basal and lower epidermal layers (Figure 2d) and p16 positivity in keratinocytes of the lower two-thirds of the epidermis (Figure 2e). Based on the patient’s history of long-term hydroxyurea therapy, the characteristic dermoscopic findings of the nailfolds and histological confirmation, a diagnosis of DM-LE/HSUD induced by hydroxyurea was made. Hydroxyurea was therefore discontinued and at the 4-month follow-up the patient showed a complete remission of the cutaneous findings on the hands and face.

## 3. Materials and Methods

**Figure 3:**
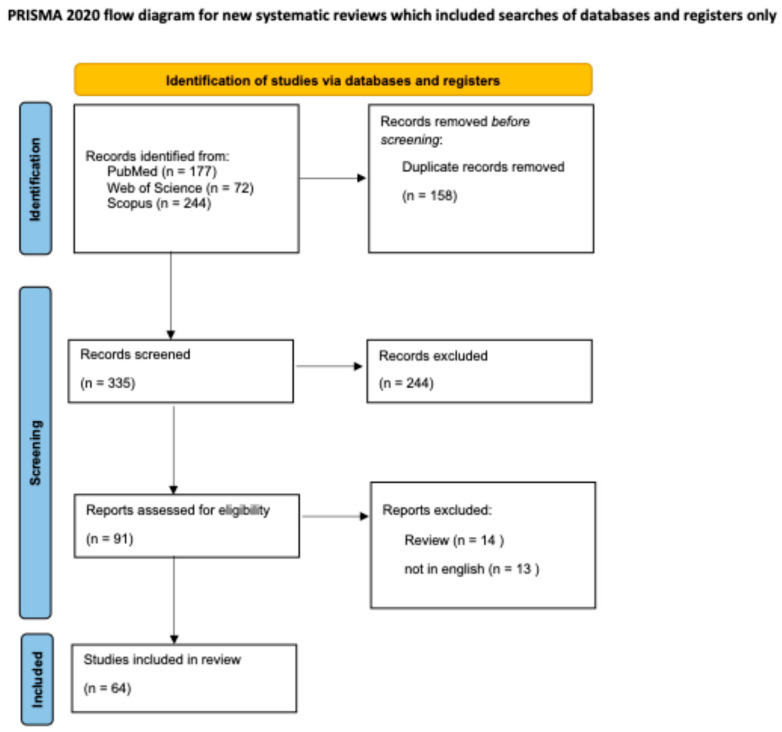
PRISMA guidelines flow-chart followed to perform this review [5].

The review was conducted in compliance with the PRISMA (Preferred Reporting Items for Systematic Reviews and Meta-Analyses) guidelines (Figure 3) [5]. We conducted a search across electronic databases, including MEDLINE (PubMed), Scopus, and Web of Science, using keywords such as “hydroxyurea-induced dermatitis”, “hydroxyurea-induced dysplasia”, “hydroxyurea-induced dermatomyositis”, and “hydroxyurea-induced squamous cell carcinoma”. We included studies published from the inception of these databases through July 2024. Abstracts were independently reviewed by two dermatologists according to predefined inclusion and exclusion criteria.

Inclusion criteria were as follows:

- Original articles, case series, and case reports on hydroxyurea-induced dermatitis, hydroxyurea-induced dermatomyositis, hydroxyurea-induced dysplasia, and hydroxyurea-induced squamous cell carcinoma.

Exclusion criteria were as follows:

- Review articles;
- Language other than English.

This systematic review was registered in PROSPERO (International Prospective Register of Systematic Reviews) Registration Number: CRD420250653625

The following information was extracted after screening: first author, year of publication, patients included, sex, age, pathology, HU exposure (years), cutaneous manifestations, histopathologic description, evolution after the discontinuation of therapy, and the presence of p53 overexpression.

## 4. Results

Then, 335 articles were screened after removing duplicates. Based on title and abstract screening, 245 studies were excluded. Therefore, 90 full texts were assessed for eligibility. From these 90 articles, dermatologists independently reviewed abstracts based on set inclusion and exclusion criteria review articles, non-English articles, were excluded, resulting in a total of 63 articles included in our review.

DM-LE

Of the 65 cases analyzed with DM-LE, 54% were female (35 patients) and 46% were male (30 patients). The mean age at diagnosis was 61.4 years (SD: ±13.61). The mean time to onset from the initiation of hydroxyurea therapy was 5.04 years (SD: ±3.23). All patients presented with lesions on their hands (100%), while the face was affected in 25% of cases (16 patients).

Histopathology revealed characteristic findings in most cases, including interface dermatitis, vacuolar alteration of basal keratinocytes, dyskeratotic keratinocytes, melanin incontinence, and vascular ectasia. Unlike classic dermatomyositis, mucin deposition was observed in only 7 cases (10.78%). Additionally, 6 patients exhibited altered expression of p53. After therapy discontinuation, among the 65 patients analyzed, 34 (52.3%) showed improvement and 8 (12.3%) died. Overall, 1 patient (1.5%) maintained a stable condition (STA), outcomes were unknown (U) in 15 cases (23.1%), and 7 (10.8%) participants continued therapy without discontinuation.

HU-Associated Squamous cell carcinoma

Among the 45 cases analyzed with HU-induced squamous cell carcinoma, 40% were female (18 patients) and 60% were male (27 patients). The mean age at diagnosis was 66.07 years (SD: ±10.10). The mean time to onset following the initiation of hydroxyurea therapy was 7.68 years (SD: ±4.15).

The lesions were predominantly located in photodistributed areas, including the face and/or scalp in 47% of cases (21 patients). The involvement of the hands was less frequent, occurring in 7 patients (6.43%). Then, 2 patients exhibited altered expression of p53.

Among the cases analyzed, clinical improvement was observed in 9 patients (20%), while no improvement was reported in 2 patients (4.4%). Unfortunately, 9 patients (20%) died following disease progression or complications.

The results are summarized in Table 1.

Table 2 and Table 3 summarize the clinical and histopathological features of the hydroxyurea-induced DM-LE (Table 2) and HUSD- and HU-associated NMSC (Table 3).

## 5. Discussion

Hydroxyurea therapy, a cornerstone in the management of myeloproliferative disorders, is associated with a spectrum of cutaneous side effects, including dermatomyositis-like eruption, hydroxyurea-associated squamous dysplasia, and nonmelanoma skin cancer, particularly squamous cell carcinoma. Our systematic review highlights the clinical features, histopathology, and outcomes of these HU-induced skin manifestations, shedding light on their potential interrelationships and clinical implications.

DM-LE commonly occurs in patients undergoing long-term HU therapy, typically appearing 25 to 121 months after initiation. Our analysis of 64 cases demonstrated that 100% of patients presented with lesions on the hands, and 25% had facial involvement. The condition clinically resembles dermatomyositis but notably lacks the associated myopathy or malignancy. Key features include xerosis, violaceous papules over the interphalangeal and metacarpophalangeal joints (Gottron’s papules-like), and, rarely, “heliotrope-like” violaceous erythema around the eyes.

Histologically, DM-LE exhibits interface dermatitis, vacuolar alteration of basal keratinocytes, dyskeratotic keratinocytes, melanin incontinence, and vascular ectasia. Mucin deposition, a hallmark of true dermatomyositis, was observed in only 10.78% of cases (7 patients). This distinction is critical, as histology alone cannot differentiate DM-LE from true dermatomyositis. Accurate diagnosis relies heavily on clinical correlation and patient history, particularly in the setting of chronic HU exposure.

Following the discontinuation of HU therapy, DM-LE generally resolves within 11 days to 19 months. Our review found that 52.3% of patients showed improvement, while others exhibited varying outcomes, including persistent lesions, stable conditions, or unknown progression. Notably, skin atrophy may persist even after resolution, highlighting the need for long-term dermatologic monitoring. While DM-LE is considered benign and typically does not necessitate HU discontinuation, its clinical resemblance to dermatomyositis raises the risk of misdiagnosis and unwarranted immunosuppression.

Chronic HU therapy is linked to more aggressive cutaneous conditions, particularly HUSD and SCC, which tend to occur in sun-exposed areas. SCC typically develops 3 to 14 years after HU initiation and is more common in individuals with Fitzpatrick skin types I and II. In our analysis of 45 SCC cases, lesions predominantly involved the face and/or scalp (47%), with hand involvement occurring in a smaller proportion of patients (6.43%).

Recent studies have introduced the term hydroxyurea-associated squamous dysplasia to describe a premalignant condition characterized by photodistributed patches of erythema and xerosis, clinically mimicking photodermatitis or DM-LE. Histologically, HUSD is marked by nuclear atypia and abnormal p53 expression, suggesting early cellular damage that can precede SCC. Interestingly, abnormal p53 expression, not typically tested, was also observed in 6 cases of DM-LE in our review, raising questions about the potential for malignant transformation.

The mechanisms underlying DM-LE-, HUSD-, and HU-induced SCC reflect the complex interplay between HU and UV radiation. Hydroxyurea induces oxidative stress and impairs DNA repair mechanisms, making keratinocytes more susceptible to UV-induced damage. This process promotes the emergence of p53-mutated clones, which can progress to dysplasia and carcinoma over time. The presence of p53 expression in DM-LE highlights a potential overlap with HUSD, suggesting that both conditions may represent stages of a chronic phototoxic process driven by long-term HU therapy and UV exposure.

Given these findings, the historical view of DM-LE as a purely benign condition warrants reevaluation. While DM-LE rarely progresses to malignancy, emerging evidence suggests that its chronicity and the presence of p53 alterations may indicate a premalignant state, particularly in patients with prolonged HU exposure. The clinical and histological overlap between DM-LE and HUSD underscores the importance of careful monitoring, rigorous photoprotection, and the consideration of HU discontinuation in high-risk patients.

## 6. Conclusions

Our findings emphasize the need for heightened vigilance in patients undergoing long-term HU therapy. Although DM-LE is typically benign and resolves after therapy discontinuation, its resemblance to true dermatomyositis and association with p53 abnormalities necessitate caution. Moreover, the potential for progression to SCC, particularly in sun-exposed areas, highlights the importance of regular dermatologic evaluations, patient education on photoprotection, and early intervention for suspicious lesions.

However, a limitation of our study is that p53 and p16 expression were not tested in the majority of included cases. Addressing this gap in future studies is essential to comprehensively assess these markers, better understand the potential premalignant nature of DM-LE, and refine clinical management strategies to mitigate the long-term cutaneous risks associated with HU therapy.

## Figures and Tables

**Table 1:** DMLE- and HU-induced SCC.

	DM-like Eruption	HU-Associated Squamous Cell Carcinoma
Number of cases	65	45
F	35	18
M	30	27
Age (mean)	61.4 (SD: +/−13.61)	66.07 (SD: +/−10.10)
Time to onset (mean)	5.04 y (SD: +/−3.23)	7.68 y (SD: +/−4.15)
Site	Hands (65 patients, 100%) and face (16 patients, 25%)	Photodistibuted: face, head and scalp in 21 patients (47%), hands in 7 patients(6.43%)
Histology	Interface dermatitis, the vacuolar alteration of basal keratinocytes, dyskeratotic keratinocytes, melanin incontinence, and vascular ectasia. Mucin, often present in true dermatomyositis, is less frequently observed, appearing in 7 patients at a rate of 10.78%..	From AK to In situ or invasive SCC
p53	6 patients	2 patients

**Table 2:** Cases reported of hydroxyurea-associated dermatomyositis-like eruption.

Authors	Sex Age	Pathology	HU Exposure (y)	Cutaneous Manifestations	Histopathologic Description	Evolution After Therapy Discontinuation
Burns et al. 1980 [6]	M/31	Chronic granulocytic leukemia	7	Thinned skin with scaly erythema on fingers, dorsal hands and toes and feet.	Hyperkeratosis, flattened epidermis, thickened granular layer, and basal layer liquefaction.	U
Burns et al. 1980 [6]	M/31	Chronic myeloid leukemia	7	Scaly erythema on fingers, dorsa of hands, toes, and soles.	NA	U
Sigal et al. 1984 [7]	F/50	Chronic myeloid leukemia	1.5	Hyperpigmentation in pressure areas, erythema and keratoses on dorsal hands/feet, bird’s beak-like nail deformities, malleolar ulcer, and tongue stomatitis with ulceration.	NA	IMP
	F/78	Chronic myeloid leukemia	1.3	Erythema and keratoses on dorsa of hands, feet, and elbows; purpuric spots on oral mucosa.	NA	IMP
	M/72	Chronic myeloid leukemia	3	Erythema on dorsa of hands evolving into ulcers; palmar erythema.	NA	NO DIS
Richard et al. 1989 [8]	F/55	Chronic myelogenous leukemia	4	Telangiectatic facial erythema; eyelid edema; scaling and atrophy on dorsa of hands; palmar keratoderma; malleolar ulcer.	Alternating epidermal atrophy and acanthosis with hyperkeratosis, cytoid bodies in the basal layer, edematous papillary dermis with melanophages, and lichenoid and perivascular mononuclear infiltrate with exocytosis. Immunofluorescence negative.	IMP
Chantereau et al. 1990 [9]	M/68	Myeloid splenomegaly	1.2	Eermatomyositis-like eruption of the hands	NA	IMP
Thomas et al. 1992 [10]	F/69	Chronic myeloid leukemia	6	Dermatomyositis-like eruption of the hands	NA	IMP
Senet et al. 1995 [11]	M/62	Chronic myelogenous leukaemia	4	Linear erythema and scaling on finger joints and dorsa of hands and feet; xerosis; palmoplantar keratoderma.	NA	IMP
	M/65	Chronic myelogenous leukaemia	10	Scaling erythema on dorsa of finger joints; purpuric plaques with necrotic leg ulcers; facial erythema.	Necrotic plaques: moderate dermal polymorphic infiltrate.	DIED
	F/59	Chronic myelogenous leukaemia	5	Scaling erythema on dorsa of finger joints; xerosis.	Hyperkeratotic epidermis, basal vacuolar changes, moderate dermal lymphocytic infiltrate, and edema.	NO DIS
	F/47	Chronic myelogenous leukaemia	9	Scaling erythema on dorsa of finger joints; palmoplantar keratoderma.	Orthokeratotic hyperkeratosis, basal vacuolar changes, moderate dermal lymphocytic infiltrate, and edema.	IMP
	F/56	Essential thrombocythaemia	2	Scaling and linear erythema on finger joints and elbows; palmoplantar keratoderma.	NA	NO DIS
	M/66	Essential thrombocythaemia	2	Scaling erythema on dorsa of finger joints; palmoplantar keratoderma; necrotic leg ulcers.	NA	IMP
Bahadoran et al. 1996 [12]	M/62	Chronic myeloid leukemia	5	Telangiectatic erythema on face, linear erythematous scaling atrophic eruption on dorsal hands, dry painful erythematous plantar keratoderma, multiple facial AKs and SCCs.	Atrophic epidermis with areas of hyperkeratosis and hypergranulosis; hydropic degeneration of the basal layer with a few Civatte bodies. Perivascular mononuclear infiltrate in the papillary dermis, extending into areas of hydropic degeneration	IMP
	M/58	Chronic myeloid leukemia	7	Multiple AKs and SCCs, diffuse skin dryness, plantar keratoderma, erythema of the hands and face	Lichenoid infiltrate in some areas; mucin deposition in the papillary dermis Direct immunofluorescence: positive IgA staining in cytoid bodies	IMP
Daoud et al. 1997 [13]	M/56–69	Chronic granulocytic leukemia	5	Poikilodermatous eruption with telangiectasia, erythema, scaling onelbows, palms and dorsal feet, and lichenoid papules on dorsal hands and fingers.	Orthokeratosis, absent granular layer, basal keratinocyte alterations, hydropic degeneration with Civatte bodies, and lichenoid lymphocytic inflammation at the dermoepidermal junction. Telangiectasia, endothelial swelling, and moderate dermal lymphocytic infiltration. DIF: vessel staining (IgG, IgM, IgA, C3) and fibrinogen deposits.	IMP
	M/56–69	Chronic myelocytic leukemia	5	Poikilodermatous eruption with telangiectasia, erythema, scaling, and lichenoid papules on the dorsal hands and fingers	Orthokeratosis, absent granular layer, basal keratinocyte alterations, hydropic degeneration with Civatte bodies, and lichenoid lymphocytic inflammation at the dermoepidermal junction. Telangiectasia, endothelial swelling, and moderate dermal lymphocytic infiltration. DIF: vessel staining (IgG, IgM, IgA, C3) and fibrinogen deposits.	IMP
	M/56–69	Chronic myelocytic leukemia	5	Poikilodermatous eruption with telangiectasia, erythema, scaling, and lichenoid papules on the dorsal hands and fingers	Orthokeratosis, absent granular layer, basal keratinocyte alterations, hydropic degeneration with Civatte bodies, and lichenoid lymphocytic inflammation at the dermoepidermal junction. Telangiectasia, endothelial swelling, and moderate dermal lymphocytic infiltration. DIF: vessel staining (IgG, IgM, IgA, C3) and fibrinogen deposits.	IMP
	F/56–69	Essential thrombocytosis	5	Poikilodermatous eruption with telangiectasia, erythema, scaling, and lichenoid papules on the dorsal hands and fingers	Orthokeratosis, absent granular layer, basal keratinocyte alterations, hydropic degeneration with Civatte bodies, and lichenoid lymphocytic inflammation at the dermoepidermal junction. Telangiectasia, endothelial swelling, and moderate dermal lymphocytic infiltration. DIF: vessel staining (IgG, IgM, IgA, C3) and fibrinogen deposits.	U
	F/56–69	Essential thrombocytosis	5	Poikilodermatous eruption with telangiectasia, erythema, scaling, and lichenoid papules on the dorsal hands and fingers	Orthokeratosis, absent granular layer, basal keratinocyte alterations, hydropic degeneration with Civatte bodies, and lichenoid lymphocytic inflammation at the dermoepidermal junction. Telangiectasia, endothelial swelling, and moderate dermal lymphocytic infiltration. DIF: vessel staining (IgG, IgM, IgA, C3) and fibrinogen deposits.	NO DIS
	F/56–69	Polycythaemia rubra vera	5	Poikilodermatous eruption with telangiectasia, erythema, scaling, and lichenoid papules on the dorsal hands and fingers	Orthokeratosis, absent granular layer, basal keratinocyte alterations, hydropic degeneration with Civatte bodies, and lichenoid lymphocytic inflammation at the dermoepidermal junction. Telangiectasia, with endothelial swelling, and moderate dermal lymphocytic infiltration. DIF: vessel staining (IgG, IgM, IgA, C3) and fibrinogen deposits.	NO DIS
Suehiro et al. 1998 [14]	F/52	Chronic myelogenous leukemia	3	Painful erythema, shallow ulcers, ichthyosis on bilateral legs and feet, facial and trunk hyperpigmentation, refractory dermatomyositis-like hand lesions evolving into ulcers.	Slight epidermal atrophy, hyperkeratosis, hypogranulosis, focal basal degeneration, hydropic degeneration, and prominent lichenoid lymphocytic inflammation. Dermal telangiectasia with and endothelial swelling, no vasculitis.	IMP
Kennedy et al. 1998 [15]	M/45	Chronic myeloid leukemia	3	Violaceous papular eruption on hands and elbow; leg and hand ulcers.	NA	IMP
	F/45	Chronic myeloid leukemia	3	Generalized hyperpigmentation especially on the back; mottled atrophic lesions resembling lichen planus on hands and heels; mild involvement on cheeks.	NA	IMP
	F/42	Chronic myeloid leukemia	4	Atrophy of the skin on the dorsum of both hands and brittle, atrophic nails	NA	NO DIS
Varma et al. 1999 [16]	F/81	Long-standing widespread psoriasis	5	Symmetrical dermatomyositis-like eruption on hands (Gottron’s papules, periungual erythema with telangiectasia, cuticular dystrophy), ulcerative lesions on legs and left foot.	Acanthosis, hyperkeratosis, focal basal degeneration, pigment incontinence, and oedematous, non-inflamed dermis. Direct immunofluorescence: positive IgM staining in cytoid bodies.	U
Vélez et al. 1999 [17]	M/42	Chronic myelogenous leukemia	3	Diffuse erythema with skin atrophy, wrinkled “cigarette paper-like” skin on dorsal hands and fingers	Diffuse and severe dermal elastosis. Immunofluorescence negative.	IMP
Vassallo et al. 2001 [18]	M/73	Chronic myeloid leukemia	2	Acral erythema, xerosis, ichthyosiform lesions, telangiectases, malleolar and oral mucosal ulcers, dermatomyositis-like changes	NA	DIED
	M/68	Chronic myeloid leukemia	0.6	Acral erythema, xerosis, ichthyosiform lesions, telangiectases, hyperpigmentation, dermatomyositis-like changes	NA	DIED
	M/57	Chronic myeloid leukemia	2	Acral erythema, xerosis, ichthyosiform lesions, telangiectases, scrotum ulcers, dermatomyositis-like changes.	NA	DIED
	M/50	Chronic myeloid leukemia	5	Acral erythema, xerosis, ichthyosiform lesions, telangiectases, scrotum ulcers, dermatomyositis-like changes, SCC (lower lip).	NA	DIED
	M/65	Chronic myeloid leukemia	5	Acral erythema, xerosis, ichthyosiform lesions, telangiectases, malleolar ulcers, keratoacanthoma (right hand), dermatomyositis-like changes.	NA	U
	F/64	Chronic myeloid leukemia	2	Acral erythema with xerosis, ichthyosiform lesions, telangiectases, stomatitis, malleolar ulcers, hyperpigmentation, dermatomyositis-like changes, and livedoid fixed erythema on heels.	NA	DIED
	F/25	Chronic myeloid leukemia	1	Acral erythema with xerosis, ichthyosiform lesions, telangiectases, hyperpigmentation, and dermatomyositis-like changes.	NA	U
Ruiz-Genao et al. 2002 [19]	M/34	Chronic myeloid leukemia pH positive	1.6	Bilateral pruritic erythema and erythematous lesions on palms, fingers and elbows.	Hyperkeratosis, focal basal degeneration with necrotic keratinocytes, and perivascular chronic infiltrate in pruritic erythematous lesions	IMP
Oskay et al. 2002 [20]	M/69	Polycythaemia vera		Dermatomyositis-like eruption on dorsal hands and face	NA	IMP
Dacey et al. 2003 [21]	F/77	Chronic myelogenous leukemia	5	Violaceous papules on knuckles, scaly erythematous plaques on lateral fingers resembling mechanic’s hands.	Mild basilar vacuolopathy, sparse perivascular lymphocytic infiltrate, and mild dermal mucin increase.	IMP
Rocamora et al. 2005 [22]	M/57	Chronic myelogenous leukemia	5	Xerosis, chronic painful ulcers, and violaceous papules/plaques of interphalangeal joints.	Slight epidermal atrophy with orthohyperkeratosis, basal vacuolar changes, colloid bodies in the papillary dermis, and slight lymphomononuclear infiltrate in the upper dermis.	STA
Yoshida et al. 2005 [23]	F/70	Polycythaemia vera	2	Scaly erythematous papules and plaques on metacarpophalangeal and proximal interphalangeal joints	Slight epidermal atrophy with hyperkeratosis, basal vacuolar changes, colloid bodies, dilated dermal vessels, no vasculitis. DIF: cytoid bodies stained positive for IgM, IgA, C3, and fibrinogen	IMP
Zaccaria et al. 2006 [24]	M/73	Essential thrombocythaemia	12	Symmetrical dermatomyositis-like eruption with pruritic poikilodermatous keratotic lesions on hands, leg ulcer, and five SCCs of the face.	NA	IMP
Elliott et al. 2006 [25]	M/53	Polycythaemia rubra vera	3	Dermatomyositis-like eruption with violaceous erythema, scaling, and atrophy on dorsal hands.	Hyperkeratosis, epidermal thinning, vacuolar changes in the basal layer with cytoid bodies. Minimal dermal lymphocytic infiltration. Autoantibodies negative.	U
Slobodin et al. 2006 [26]	F/57	Chronic myeloid leukemia	5	Dry, scaly, red-violaceous plaques on hands and forearms, particularly on dorsal MCP and PIP joints (Gottron’s papules).	NA	DIED
Haniffa et al. 2006 [27]	F/52	Polycythaemia rubra vera	5	Painful leg ulcers, widespread telangiectases, violaceous facial erythema, and purple interphalangeal papules.	Epidermal atrophy, basal cell hydropic degeneration with cytoid bodies and dyskeratotic cells, and mild perivascular lymphocytic infiltrate.	U
Martorell-Calatayud et al. 2009 [28]	F/76	Essential thrombocythaemia	5	Leg ulcers and non-pruritic scaly erythematous lesions of interphalangeal joints.	Mild hydropic degeneration of the basal layer, slight lymphocytic infiltration in the superficial and mid-dermis, and moderate interstitial mucin deposition.	IMP
Janerowicz et al. 2009 [1]	M/74	Polycythaemia rubra vera	2	Intense xerosis (ichthyosis-like), violaceous papules on dorsal hands, and heliotrope-like periorbital erythema.	Thin, atrophic epidermis with Civatte bodies and sparse perivascular mixed dermal infiltrate.	IMP
Kalajian et al. 2010 [2]	F/82	Myelodysplastic syndrome		Gottron’s papules, scaly erythematous plaques on hands, periungual erythema, confluent facial erythema, xerosis, poikiloderma, leg ulcer.	Lichenoid inflammation with basal vacuolar changes, occasional Civatte bodies, apoptotic keratinocytes, and mild superficial dermal lymphocytic infiltrate. Focal p53 expression in a confluent nuclear pattern along the lower levels of the epidermis.	DIED
Cook-Norris et al. 2010 [29]	F/62	Myelodysplasia	5	Pruritic eruption of the dorsum of her hands. shiny, violaceous papules over herknuckles and erythematous, reticulated, scaly plaques onher fingers and the dorsum of her hands, mimick-ing Gottron papules and mechanic hands of dermatomyosi-tis.	Hyperkeratosis and epidermalatrophy. The basal layer showed vacuolar change and cytoid bodies.	IMP
Agrawal et al. 2012 [30]	F/45	Chronic myelogenous leukemia	5	Shiny, scaly erythematous papules and plaques on MCP and PIP joints of hands.	Slight epidermal atrophy, orthohyperkeratosis, basal vacuolar changes, colloid bodies, melanophages, and slight perivascular lymphomononuclear infiltrate.	NO DIS
Nofal et al. 2012 [31]	F/68	Chronic myeloid leukemia	6	Gottron’s papules, atrophy, xerosis, ichthyosis, photosensitivity, hyperpigmentation (skin, oral, nail), acral erythema, palmoplantar keratoderma, actinic keratoses on hands, and leg ulcers.	Epidermal atrophy with hyperkeratosis, vacuolar degeneration in the basal layer, and mononuclear perivascular infiltrate in the upper and mid-dermis.	IMP
Zappala et al. 2012 [32]	F/66	Myelofibrosi	5	Violaceous keratotic lesions oh the hands with a painful lateral malleolus ulcer.	Lichenoid inflammation with basal vacuolar changes, occasional Civatte bodies, apoptotic keratinocytes, and mild superficial dermal lymphocytic infiltrate.	IMP
De Unamuno-Bustos et al. 2014 [33]	F/76	Idiopathic myelofibrosis	4	Desquamating erythematous plaques over interphalangeal joints, periorbital erythema, erythematous papular frontal and retroauricular lesions, and malleolar ulcer with atrophic erythematous border.	Acanthotic epidermis with hyperkeratosis, dyskeratosis, basal vacuolization, and lichenoid interface dermatitis. Keratinocyte disorganization and atypia with large nuclei. Intense expression of p53 in the dysplastic keratinocytes	IMP
Ito et al. 2014 [34]	M/69	Essential trhombocytosis		Amyopathic dermatomyositis with Gottron’s papules and heliotrope erythema.	NA	U
Koch et al. 2016 [35]	F/84	Polycythaemia vera	4.5	Periorbital telangiectatic erythema; scaly plaques on cheeks, forehead, and hands; xerosis; small leg ulcers near malleoli.	Basal vacuolar degeneration with necrotic keratinocytes and mild lymphohistiocytic infiltrate in the superficial dermis.	IMP
Moreno-Artero et al. 2017 [36]	F/63	Essential thrombocythaemia	6	Erythematous, scaly lesions on hands, feet, elbows, knees, and presternal area.	NA	IMP
Calleja Algarra et al. 2017 [37]	M/45	Essential thrombocytosis	1	Purplish-red, infiltrated plaques symmetrically on proximal interphalangeal joints; pronounced palmoplantar hyperkeratosis.	NA	U
Marie et al. 2017 [38]	F/68	Chronic myelogenous leukemia	7	Dermatomyositis-like band-like scaling erythema on the hands, periorbital erythema with heliotrope rash, painful pretibial leg ulcer.	Hyperkeratotic epidermis with basal vacuolar changes and moderate pericapillary lymphocytic infiltrates. No granulomas or vasculitis. Direct immunofluorescence negative.	IMP
Platto et al. 2018 [39]	F/69	Essential trhombocytosis		Violaceous plaques on dorsal hands and interphalangeal joints; enlarging, painful leg ulcer.	Epidermal atrophy, vacuolar interface dermatitis with dyskeratotic keratinocytes, and perivascular lymphocytic infiltrates.	IMP
Veraitch et al. 2019 [40]	M/40	Polycythaemia vera	0.75	Erythematous pigmented flat-topped papules on the face, forehead and eyelids, and on the elbows and fingers.	Acanthotic epidermis with interface dermatitis and scattered basal necrotic keratinocytes. Mild perivascular lymphocytic infiltrate in the superficial papillary dermis with melanophages.	IMP
Pruessmann et al. 2024 [41]	M/64	Chronic myeloid leukemia	12	Atrophic, poikilodermatous, scaly patches on dorsa of hands with accentuation over proximal interphalangeal joints; similar palmar and plantar lesions.	Acanthosis, orthokeratosis, atrophy of the epidermidis; pigment incontinence; vascular proliferation and fibrosis.	U
	F/85	Polycythemia vera	10	Erythematous macules on décolleté; worsening scaly plaques on arms; erythematous papules on dorsa of hands.	Acanthosis, orthokeratosis and parakeratosis, atrophy of the epidermidis; pigment incontinence; interface dermatitis; vascular proliferation and fibrosis. Suprabasal p53 expression.	U
	M/73	Polycythemia vera	12	Erythematous papules on dorsa of hands, accentuated on PIP joints.	Acanthosis, parakeratosis; interface dermatitis. vascular proliferation and fibrosis. Suprabasal p53 expression.	U
	F/73	Myeloproliferative neoplasm	16	Erythema and papules on face; scaly erythematous papules and plaques on hands, accentuated on PIP joints.	Acanthosis, parakeratosis; pigment incontinence. interface dermatitis; vascular proliferation and fibrosis. Suprabasal p53 expression.	U
Ahdoot et al. 2024 [42]	F/84	Myeloproliferative neoplasm	12	Violaceous scaly, atrophic thin plaques on her dorsal hands	Subtle vacuolar interface dermatitis with a mild increase in dermal mucin and vascular ectasia.	U
Our case	F/70	Polycytemia vera	12	Gottron’s papules on the dorsa of her hands, erythematous rash on the face	Orthokeratotic hyperkeratosis with slight epidermal atrophy, superficial angioplasia, mild perivascular lymphocytic infiltrate; occasional mucin deposits, keratinocyte atypia, dyskeratosis and nuclear irregularities p53 positivity in the basal and lower epidermal layers; p16 positivity in keratinocytes of the lower two-thirds of the epiderm.	IMP

Legend: AK = actinic keratosis; DIED = died; DIF = direct immunofluorescence; IMP = improvement; NA = nonavailable; NO DIS = no discontinuation; STA = stable; SCC = squamous cell carcinoma; U = unknown.

**Table 3:** Cases reported of hydroxyurea-associated squamous dysplasia and hydroxyurea-associated nonmelanoma skin cancer.

Authors	Sex /Age	Pathology	HU Exposure (Years)	Cutaneous Manifestations	Histopathologic Description	Evolution After Therapy Discontinuation
Disdier et al. 1991 [43]	M/65	Chronic granulocytic leukemia	2	Multiple SCCs of the head, scalp, hands; multiple AK, BCC, SCC, thin pellagroid-like skin	Infiltrating SCCs	Switch from HU to Mercaptopurine. Vertex lesion reoccured after surgical excission, infiltrating the skull and with metastatic cervical lymphnodes. Died.
Papi et al. 1993 [44]	M/70	Chronic myeloid leukemia	4	Leg ulcer, multiple AK, SCC (left temple), BCC; diffuse xerosis, DM-like erythema and atrophy hands	Severe orthokeratotic hyperkeratosis of the hands lesions, cells containing mucin bodies	Switch from HU to Etretinate and Busulfan. Surgical excision of SCC. After few weeks other SCC appeared on the head while dermatomyositis-like lesions of the hand and elbows slowly worsened.
Angeli-Besson et al. 1995 [45]	M/67	Chronic myeloid leukemia	4	Multiple SCC (adenoid type) of the scalp, cutaneous horn of the left cheeck, solar elastosis of the rest of the face	Squamous-cell carcinomas with adenoid feature structures.	Switch from HU to Mercaptopurine. Splenectomy.
Grange et al. 1995 [46]	F/60	Chronic myeloid leukemia	6	Rapid, multiple infiltrative AKs, xerosis, melanonychia; lichenoid papules and atrophy of the hands	NA	NA
Bahadoran et al. 1996 [12]	M/62	Chronic myeloid leukemia	5	Painful keratoderma, telangiectatic erythema, linear erythematous scaling athrophic eruption, multiple facial AKs and SCCs	Atrophic skin. In some areas, hyperkeratosis and hypergranulosis. Moderate hydropic degeneration of the basal layer and a few Civatte bodies. In the papillary dermis, perivascular mononuclear infiltrate extending to the overlying epidermis in the areas of hydropic degeneration.	Switch from HU to Busulfan. Resolution after a few months.
	M/58	Chronic myeloid leukemia	7	Multiple AKs and SCCs of the face, diffuse skin dryness, plantar keratoderma, erythema of the hands and face	Lichenoid infiltrate in some areas. Colloidal iron and alcian blue stains showed mucin deposition in the papillary dermis. DIF showed positive staining of the cytoid bodies for IgA.	Moderate improvement after discontinuation.
Callot-Mellot et al. 1996 [47]	M/64	Essential thrombocythemia	5.5	3 SCCs and AKs of the scalp; xerosis, melanonychia	NA	Discontinuation of HU. No reoccurrence in 3 years follow up.
	M/72	Polycythaemia rubra vera	7	5 BCC, AKs	NA	Discontinuation of HU. No reoccurrence in 2.5 years follow up.
	F/69	Polycythaemia rubra vera	8.5	SCC of the hand, AKs, plantar keratoderma, erythema	NA	No withdrawal. No reoccurrence
	F/76	Essential thrombocythemia	10	BCCs, AKs, nycholysis and alopecia	NA	Unavailable for follow up
	F/76	Essential thrombocythemia	2	3 BCCs, AKs	NA	Unavailable for follow up
De Simone et al. 1998 [48]	M/59	Chronic myelogenous leukemia	6	Multiple nodular lesions of the head (SCC and AK)	SCC and AK of the head	Switch from HU to Busulfan.
Best et al. 1998 [49]	F/59	Essential thrombocythaemia	6.5	Scaly red patch of the face, facial telangiectasia, dry white scaling of her palms and soles, and pink-red flat papules over the bony prominences of her hands. 4 SCC (3 of the face, 1 of the hand)	3 SCCs in situ of the face, 1 SCCs of the hand with dermal mucinosis, telangiectasia and solar elastosis. 3 BCCs and 10 Aks.	Switch from HU to Anagrelide
	F/50	Chronic myelogenous leukemia	0.5	Multiple ulcers, BCC of the left nasal ala, SCC of the lip	BCC of the left nasal ala, SCC of the lip	Switch from HU to interferon-alpha. Resolution of leg ulcers in 8M. 4Y after discontinuation multiple SCC of the hands developed. Died that year.
Young et al. 2000 [50]	F/72	Polycythaemia rubra vera	.	Poikilodermatous eruption with ichtiosys and lichenoid papules oh her cheeks and forehead; longitudinal bands of pigmentation of her toe and thumb nails. Non-scarring alopecia, finger nail dystrophy. AKs of the face and forehead. SCC of the forehead.	SCC	Switch from HU to interferon-alpha, slow improvement of lesions is observed. Cryotherapy for AK.
Salmon-Ehr et al. 2000 [51]	M/73	Polycythaemia rubra vera	10	AKs, SCCs (ear)	NA	Switch from HU to Busulfan. Surgical excission of SCC. No reoccurrence in 14 months follow-up.
Aste et al. 2001 [52]	M/60	Chronic myeloid leukemia	10	SCCs on the face, scalp and back of his hands.	NA	Switch from HU to Mercaptopurine combined with Etretinate for 3 months, without improvement. Chemotherapy based on Vincristine on day 1 followed by Bleomycin for the next 4 days, but only mild clinical improvement.
Vassallo et al. 2001 [18]	M/50	Chronic myeloid leukemia	5	Acral erythema, xerosis, ichthyosiform lesions, telangiectasias, scrotum ulcers, dermatomyositis-like changes, squamous-cell carcinoma (lower lip).	Hyperkeratosis, irregular acanthosis, sparse superficial perivascular lymphohistiocytic infiltrate with focal dermo-epidermal interface changes and, rarely, dyskeratosis of single keratinocytes. Inflammatory infiltarte, dilatated blood vessels and atrophy of the erythematous areas.	Switch from HU to Pentoxyfilline. Died.
	M/65	Chronic myeloid leukemia	5	Acral erythema, xerosis, ichthyosiform lesions, telangiectasias, malleolar ulcers, keratoacanthoma (dorsum of right hand), dermatomyositis-like changes	Hyperkeratosis, irregular acanthosis, a sparse superficial perivascular lymphohistiocytic infiltrate with focal dermo-epidermal interface changes and, rarely, dyskeratosis of single keratinocytes. Inflammatory infiltarte, dilatated blood vessels and atrophy of the erythematous areas.	Switch from HU to Pentoxyfilline
	M/55	Chronic myeloid leukemia	7	Acral erythema, ichthyosiform lesions, lower-lip ulcer, glossitis, squamous-cell carcinoma (left ear)	Hyperkeratosis, irregular acanthosis, a sparse superficial perivascular lymphohistiocytic infiltrate with focal dermo-epidermal interface changes and, rarely, dyskeratosis of single keratinocytes. Inflammatory infiltarte, dilatated blood vessels and atrophy of the erythematous areas.	No treatment. Died.
	M/70	Chronic myeloid leukemia	5.75	Acral erythema, xerosis, ichthyosiform lesions, telangiectasias, squamous-cell carcinoma (forehead), livedoid fixed erythema of heels.	Hyperkeratosis, irregular acanthosis, a sparse superficial perivascular lymphohistiocytic infiltrate with focal dermo-epidermal interface changes and, rarely, dyskeratosis of single keratinocytes. Inflammatory infiltarte, dilatated blood vessels and atrophy of the erythematous areas.	No treatment. Died.
	F/64	Chronic myeloid leukemia	10	Acral erythema, xerosis, ichthyosiform lesions, malleolar ulcers, keratoacanthoma (left cheek), hyperpigmentation.	Hyperkeratosis, irregular acanthosis, a sparse superficial perivascular lymphohistiocytic infiltrate with focal dermo-epidermal interface changes and, rarely, dyskeratosis of single keratinocytes. Inflammatory infiltarte, dilatated blood vessels and atrophy of the erythematous areas.	Switch from HU to Pentoxyfilline. Died.
Esteve et al. 2001 [53]	F/83	Polycythaemia rubra vera	13	5 photodistributed SCCs, 2 oral SCCs	NA	NA
Pamuk et al. 2003 [54]	M/73	Chronic myeloid leukemia	3	Scaly lesion, redness and dryness on his left posterior auricle	SCC metastatic to the parathyroid gland and regional lymph nodes	Patient refused to change drug so HU intake was reduced. 1Y after SCC was metastatic. Surgery (ear grafting and radical neck and lymph nodes dissection) and radiotherapy. HU suspended after surgery, but patient died few weeks after.
Sanchez-Palacios et al. 2004 [4]	M/69	Essential thrombocythemia	9	8 squamous cell carcinomas and multiple diffuse hypertrophic AKs on sunexposed areas	Diffuse atypia of the epidermis with disarray of keratinocytes, and acantholysis and focal vacuolar changes of the lower layers of the epidermis. Diffuse expression of p53 along the lower layers of the epidermis. Supportive of the dysplastic nature of the acantholytic process.	NA
	M/66	Chronic myelogenous leukemia	11	2 SCCs, 1 BCC and multiple hypertrophic AKs on his scalp; malleolar ulcer	NA	NA
De Benedittis et al. 2004 [55]	M/66	Polycythaemia vera	15	Alopecia, dryness, erythema, atrophy, skin and nail hyperpigmentation, ulcer of the left leg. White ulcerative lesion (SCC) of the left margin of the toungue.	Moderately differentiated oral SCC (T2N0M0)	Switch from HU to Thioguanine, Pentoxyfilline. Treatment of SCC with hemiglossectomy, hemimandibulectomy, removal of the salivary glands and latero-cervical lymphnodes.
Zaccaria et al. 2006 [24]	M/73	Essential thrombocythaemia	NA	Itchy cutaneous eruption. Leg ulceration. Poikilodermatous keratotic lesions of the dorsum of hands, face and back (5 SCCs).	Well-differentiated SCC	Switch from HU to Busulfan. Complete resolution of DM-like lesions and normal platelet count in 90 days.
Kalajian et al. 2010 [2]	F/82	Myelodysplastic syndrome		Gottron’s papules, scaly erythematous plaques on hands, confluent facial erythema, xerosis, poikiloderma, leg ulcer. SCC in situ of the dorsal right index.	Lichenoid inflammation with basal vacuolar changes, occasional Civatte bodies, apoptotic keratinocytes and mild superficial dermal lymphocytic infiltrate.	SCCs continued to progress over the next 3 months. Died 5 months later from complications of her myelodysplastic syndrome.
Schleußinger TM et al. 2011 [56]	F/80	Essential thrombocytosis	13	Multiple large hyperkeratotic lesions (AKs and SCCs in situ) of the face	Bowenoid AK and Bowen disease.	NA
Stone et al. 2011 [57]	F/62	Polycythaemia vera	9	Painful non healing chronic ulcer on the left heel.	Invasive SCC at the ulcer area with tumour cells characterized by enlarged nuclei with angulated contours and prominent nucleoli.	No improvement (treatment with HU was stopped 2Y before consultation). Surgery for SCC. Aspirin for polycythemia vera.
Radić et al. 2011 [58]	F/76	Polycythaemia vera	NA	BCC and SCC on the dorsum of the hand	BCC and poorly differentiated sarcomatoid SCC (positive for vimentin, EMA and cytokeratins CK5/6, and negative for bcl-2). p53 was positive in approximately 50% of squamous cell carcinoma cells and in almost all basal cell carcinoma cells	NA
Neill et al. 2013 [59]	M/67	Polycythaemia vera	11	Extensive photodamage, atrophy, and dermatoheliosis on the dorsal hands and posterior forearms; hyperkeratotic papule on his right helix (SCC); dermatomyositis-like hypopigmented pink papules (Gottron’s papules) and plaques on hands.	Invasive SCC of the helix, AK of the hands, right fifth proximal interphalangeal joint showed sparse lymphocitic infiltrate and necrotic keratinocytes compatible with DM.	Improvement over a month of discontinuation from HU.
Antar et al. 2014 [60]	F/60	Essential thrombocythaemia	5	Painful erythematous ulcerated plaques bilateral on feet.	Infiltrating and keratinizing SCC, with areas of SCC in situ.	Switch from HU to Anagrelide (non tolerated), then Peginterferon and subsequent improvement. Debridement and grafting of the ulcerated lesions
Uday Kumar et al. 2017 [61]	M/30	Chronic myeloid leukemia	5	Diffuse ulceroproliferative lesion of the buccal mucosa (SCC), and mucositis.	Hydroxyurea-induced erosive lichen planus of oral cavity	Discontinuation from HU. Radiotherapy for SCC. Buccal lesion did not re-occurred. Died after 1 year.
Cantisani et al. 2019 [62]	9 patients. Mean age: 77 ± 6.3 years, 1F, 8M	Polycythaemia vera	NA	AKs in 7 patients on the head; among these, one patient also developed a BCC, one patient developed a SCC, and one patient developed both a SCC and a BCC. The other two patients of our cohort developed solely BCC on the trunk and on the head.	NA	Daylight photodynamic therapy to treat NMSC, Imiquimod in one patient who had multiple ulcerated lesions.
Xu et al. 2019 [63]	F/67	Primary myelofibrosis	20	Nodular and keratotic lesions with sharp margins, branny desquamation, hyperpigmentation on her dorsal hands and legs, and ulcerative lesions (SCC) on her left ankle	Well-differentiated SCC of the ulcerated ankle with abundant cytoplasmic keratin pearls	Radical surgery with under-the-knee amputation and preventive right groin nodal dissection. Healing with scabbing of the hands’ wounds.
Brown et al. 2020 [64]	F/84	Essential thrombocythaemia	17	Multiple hyperkeratotic lesions, AKs and SCCs on her right forearm, left hand, right chin.	Intra-epidermal SCC	Hydroxyurea suspended. Impossible complete excision of SCC. Palliative radiotherapy and topical 5FU and Acitretin.
Kerdoud et al. 2021 [65]	F/59	Essential thrombocytosis		Painless slow-growing nodular lesion, black horn-shaped hyperkeratosis lesion of the nasal pyramid	Well-differentiated SCC	Switch from HU to Busulfan. Maxillofacial surgery of SCC

Legend: AK = actinic keratosis; BCC = basal cell carcinoma; DIF = direct immunofluorescence; NA = nonavailable; SCC = squamous cell carcinoma.

## Data Availability

Data are contained within the article.
